# Interaction between Nitrogen and Phosphate Stress Responses in *Sinorhizobium meliloti*

**DOI:** 10.3389/fmicb.2016.01928

**Published:** 2016-11-30

**Authors:** Kelly L. Hagberg, Svetlana N. Yurgel, Monika Mulder, Michael L. Kahn

**Affiliations:** ^1^School of Molecular Biosciences, Washington State University, PullmanWA, USA; ^2^Institute of Biological Chemistry, Washington State University, PullmanWA, USA

**Keywords:** nitrogen stress, phosphate stress, glutamine synthetase, alkaline phosphatase, P_II_ nitrogen regulatory proteins, Phob

## Abstract

Bacteria have developed various stress response pathways to improve their assimilation and allocation of limited nutrients, such as nitrogen and phosphate. While both the nitrogen stress response (NSR) and phosphate stress response (PSR) have been studied individually, there are few experiments reported that characterize effects of multiple stresses on one or more pathways in *Sinorhizobium meliloti*, a facultatively symbiotic, nitrogen-fixing bacteria. The P_II_ proteins, GlnB and GlnK, regulate the NSR activity, but analysis of global transcription changes in a P_II_ deficient mutant suggest that the *S. meliloti* P_II_ proteins may also regulate the PSR. P_II_ double deletion mutants grow very slowly and pseudoreversion of the slow growth phenotype is common. To understand this phenomenon better, transposon mutants were isolated that had a faster growing phenotype. One mutation was in *phoB*, the response regulator for a two component regulatory system that is important in the PSR. *phoB*::Tn*5* mutants had different phenotypes in the wild type compared to a P_II_ deficient background. This led to the hypothesis that phosphate stress affects the NSR and conversely, that nitrogen stress affects the PSR. Our results show that phosphate availability affects glutamine synthetase activity and expression, which are often used as indicators of NSR activity, but that nitrogen availability did not affect alkaline phosphatase activity and expression, which are indicators of PSR activity. We conclude that the NSR is co-regulated by nitrogen and phosphate, whereas the PSR does not appear to be co-regulated by nitrogen in addition to its known phosphate regulation.

## Introduction

*Sinorhizobium meliloti* are important bacteria due to their ability to fix atmospheric nitrogen during symbiosis with alfalfa and other legumes. Nitrogen availability is a common problem that farmers need to address in order to approach the yield potential of many crops. The application of nitrogen fertilizers to the soil is a common solution (Ladha et al., 2005; Hirsch and Mauchline, 2015). Using fertilizers is expensive and their use is also associated with significant environmental costs (Ladha et al., 2005; Spiertz, 2010; Hirsch and Mauchline, 2015). A significant fraction of the fertilizer typically runs off farmland and contaminates groundwater and river ecosystems. Microbial transformation of nitrogen fertilizers can lower soil pH, and affect crops that do not have the ability to compensate for the increased acidity. One way to reduce the need for nitrogen fertilizer applications is to grow legumes that can form symbiotic relationships with rhizobia, allowing biological nitrogen fixation (BNF) to occur *in situ* and on demand. In the process of establishing a symbiosis with a legume like alfalfa, nodules are formed on the roots after the bacteria infect emerging root hairs. In these nodules, bacteria invade plant cells and differentiate into an organelle-like state called a bacteroid. The bacteroids produce nitrogenase and associated proteins, which reduce atmospheric dinitrogen into ammonia, a process termed BNF. The plant can use the ammonia for incorporation into amino acids, nucleic acids, and other compounds necessary for plant survival (Patriarca et al., 2004; Oldroyd and Downie, 2008). In order to support nitrogen fixation, plants supply the bacteroids with carbon compounds and the nodule maintains a microaerobic environment that is compatible with bacterial respiration and nitrogen fixation. Establishing a symbiotic relationship is facultative for both the plant and bacteria and nodules are formed *de novo* in response to the plant’s need for nitrogen.

For BNF to occur and provide useable nitrogen to legumes, free-living rhizobia must be present in the soil and the bacteria therefore need to be able to survive in various soil conditions, adapting to nutrient limitations, soil acidity and moisture content. Soil nutrient availability can be dynamic, with the availability of nutrients shifting as the season and presence of other microorganisms changes (Vitousek et al., 2010; Fanin et al., 2016). Rhizobia have stress response pathways to deal with numerous environmental limitations and help them persist in the rhizosphere (Al-Niemi et al., 1997; Krol and Becker, 2004; Yurgel et al., 2012). Nutrient stress responses alter gene expression and protein activity to improve uptake and catabolism of substrates that contain phosphorus or nitrogen, more efficiently incorporate the compounds into metabolism and, in some situations, use biosynthetic pathways that are not as dependent on these nutrients (Battesti et al., 2011). The nitrogen and phosphate stress responses (PSRs) in *S. meliloti* have each been characterized. While certain aspects may not be entirely understood yet, a fair amount of detail has been elucidated for each pathway.

**FIGURE 1:**
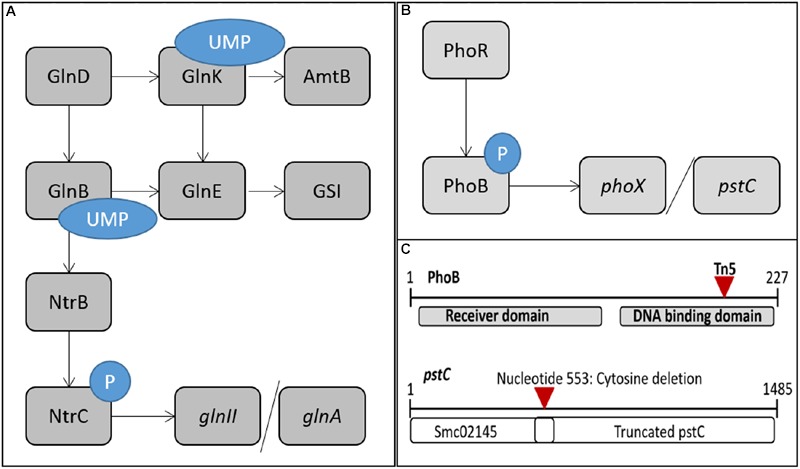
**The NSR and PSR are used by *Sinorhizobium meliloti* to cope with nitrogen and phosphate limitations.** Cartoon models of the regulatory circuits of the NSR and PSR are depicted here, as well as the *phoB*::Tn*5* mutation, and *pstC* repair. **(A)** Model of the NSR during low nitrogen conditions. In response to low nitrogen, GlnD uridylylates GlnB and GlnK. GlnK-UMP activates the ammonium transporter AmtB. Both GlnB-UMP and GlnK-UMP act on GlnE to deadenylylate GSI-AMP and produce the active form of GSI. GlnB-UMP activates the NtrB/NtrC two-component system. NtrB phosphorylates NtrC. NtrC-P binds target promoters to activate a set of genes, including *amtB, glnII*, and *glnA.* **(B)** Model of the PSR during low phosphate conditions. In response to low levels of phosphate, PhoR phosphorylates PhoB. PhoB-P binds target promoters, such as *phoX* and *pstC*, to activate gene expression. **(C)** Cartoon depicting the site of Tn*5* transposon insertion in PhoB and the mutation in *pstC* leading to the dysfunctional Pst transport system in *S. meliloti* Rm1021.

During nitrogen limitation, the nitrogen stress response (NSR) in *S. meliloti* is active (**Figure 1A**). The NSR increases uptake and mobilization of nitrogen while increasing the efficiency with which available nitrogen is used (Merrick and Edwards, 1995; Arcondeguy et al., 2001; Leigh and Dodsworth, 2007). Regulation of the core NSR in *S. meliloti* involves a sensor protein, GlnD, two P_II_ proteins, GlnB and GlnK, and the NtrB/NtrC two component regulatory system. GlnD, a uridylyltransferase/uridylyl-cleavage enzyme, senses the ratio between glutamine and alpha-ketoglutarate, an index of nitrogen availability (Merrick and Edwards, 1995; Ikeda et al., 1996; Jiang et al., 2012). When alpha-ketoglutarate levels are high, indicating nitrogen deficiency, GlnD uridylylates GlnB and GlnK. GlnB-UMP and GlnK-UMP act on GlnE which regulates the adenylylation state of glutamine synthetase I (GSI) (Atkinson and Ninfa, 1998). GSI-AMP is inactive and when the adenylyl group is removed, GSI becomes activated allowing it to add ammonium to glutamate to form glutamine, a molecule which is used to donate nitrogen in many cellular biosynthetic reactions (Yurgel et al., 2010). In addition to influencing GlnE activity, GlnK regulates the AmtB transporter (Gruswitz et al., 2007). When nitrogen is low, GlnK-UMP acts on AmtB to increase uptake of nitrogen from the environment. GlnB-UMP acts on the two-component NtrB/NtrC system by allowing the NtrB sensor protein to be phosphorylated during low nitrogen conditions. NtrB-P then transfers the phosphate group to the response regulator, NtrC. NtrC-P increases transcription of target genes through DNA binding and interaction with RpoN, a sigma54 subunit of RNA polymerase. Genes activated by NtrC-P are primarily involved in nitrogen uptake and mobilization. One such target is *glnII*, coding for a second glutamine synthetase, GSII. *glnII* expression is often used as an indicator of NSR activity (Arcondeguy et al., 1997) due to its direct activation by NtrC-P.

Deletion of the P_II_ proteins results in slow growing, small colonies in *S. meliloti* (Yurgel et al., 2010). Poor growth in strains lacking the P_II_ proteins is also observed in *Rhodospirillum rubrum* (Zhang et al., 2001), *Escherichia coli* (minimal media only) (Atkinson and Ninfa, 1998), *Azospirillum brasilense* (de Zamaroczy, 1998), and *Azorhizobium caulinodans* (Michel-Reydellet and Kaminski, 1999). Spontaneous reversion of the growth rate in these strains does occur, a phenomena known as pseudoreversion. In addition to poor growth, P_II_ deletion strains consistently display increased GS activity. Suppressor mutations were identified in *R. rubrum* that restore growth of a P_II_ deletion strain (Zhang et al., 2006). The mutations were found in five distinct genes and in each case, total GS activity was decreased compared to the parent strain, leading to the conclusion that excessive GS activity is leading to the slow growth phenotype of P_II_ deletion strains. In *S. meliloti*, the Rm1021*ΔglnBΔglnK* deletion strain (hereafter referred to as BK) displays increased GS activity compared to wild type 1021 (Yurgel et al., 2010), but there are no published studies relating the growth rate to GS activity in BK pseudorevertants.

In the rhizosphere, both phosphate and nitrogen are often limiting nutrients. *S. meliloti* engage a PSR to cope with phosphate limitation (**Figure 1B**) (Al-Niemi et al., 1997). The PSR involves fewer regulatory and sensor proteins than the NSR, with the core regulation carried out by a two-component regulatory system, PhoR/PhoB. In this system, PhoR is the sensor kinase that detects the phosphate status of the cell. When phosphate supply is limited, PhoR becomes phosphorylated and then transfers the phosphate to PhoB, the response regulator. PhoB-P then regulates transcription of its target genes by binding in promoter regions where it interacts with RpoD, the sigma 70 subunit of RNA polymerase (Makino et al., 1993; Al-Niemi et al., 1997). PhoB regulated genes include those involved in phosphate uptake, transport and metabolism as well as exopolysaccharide production and cell protection genes (Mendrygal and Gonzalez, 2000; Yuan et al., 2006a). Alkaline phosphatases (AP) like PhoX have increased expression and activity during phosphate stress enabling the bacteria to release phosphate from environmental compounds and make it available for transport into the cell (Zaheer et al., 2009).

Transport of phosphate is complicated and involves several different transport systems that differ in their capacity and affinity. The high affinity, high velocity *pst* transporter is one of the primary transport systems for phosphate uptake in *S. meliloti*. Rm1021, a strain that is commonly used when conducting research on *S. meliloti*, has a defective *pstC* gene that produces a truncated protein (**Figure 1C**) (Yuan et al., 2006a). This leads to defective transport, resulting in constitutive activation of PhoB (Yuan et al., 2006a) and a moderate level of PSR activation at all times. In the course of this work, the *pstC* gene mutation was repaired and experiments were performed in both wild type Rm1021 and a strain of Rm1021 with the repaired allele, which has been designated *pstC+.* This allowed us to relate previous studies performed in Rm1021 (Yuan et al., 2006a; Yurgel et al., 2010, 2013) with studies done here and in other strains having a properly functioning PSR (Arcondeguy et al., 1997; Krol and Becker, 2004).

The NSR and PSR pathways have been characterized independently and only recently has data been presented indicating regulatory crosstalk (Krol and Becker, 2004; Martín et al., 2011; Yurgel et al., 2013). It is accepted that *glnII* is regulated by the NtrB/NtrC system, but the expression of *glnII* has been shown to be dependent on environmental phosphate conditions as well (Krol and Becker, 2004). Additionally, a change in expression of phosphate-related genes during nitrogen stress was described leading to more evidence of crosstalk between these two stress responses (Yurgel et al., 2013). We hypothesized that there was a coordination of the NSR and PSR and that phosphate conditions may also regulate aspects of the NSR, such as GS expression or activity. It was also hypothesized that nitrogen may affect aspects of the PSR, such as AP expression or activity. In this paper, we characterize a *phoB*::Tn*5* mutant that alters the phenotypes of a P_II_ deletion strain and lends support to crosstalk between the regulatory systems. We also demonstrate that phosphate conditions affect GS expression and activity, but nitrogen does not affect AP expression and activity in Rm1021 strains.

## Materials and Methods

### Strains, Plasmids, and Media

Strains and plasmids used are listed in **Table 1**. *S. meliloti* was grown at 30°C minimal mannitol NH_4_Cl or YMB liquid medium or medium that had been solidified with 1.5% agar (Sigma) (Somerville and Kahn, 1983). For assays using high or low nitrogen and/or phosphate, Min MOPS (Al-Niemi et al., 1997) liquid media containing 0.4% NH_4_Cl (nitrogen-sufficient) or 0.04% glutamate (nitrogen-deficient) was used. KH_2_PO_4_ was added at 2 mM for phosphate sufficient conditions and 50 μM for phosphate deficient conditions (Bardin et al., 1996). *E. coli* strains were grown at 37°C on LB media (Sambrook, 2001). Neomycin was added at 200 μg/mL and tetracycline at 10 μg/mL for *S. meliloti*. For *E. coli*, kanamycin was added at 40 μg/mL. LBMC was used for transduction (Finan et al., 1984). 5-Bromo-4-chloro-3-indolyl phosphate (X-Phos) was added to solid media at a concentration of 50 μg/mL.

**Table 1:** Strains and plasmids.

Strain or plasmid	Genotype or characteristic	Source/reference
*Sinorhizobium meliloti* Rm1021	Wild type	Galibert et al., 2001
Rm2011	Wild type	T. Huguet laboratory
Rm1021Δ*glnB*Δ*glnK*	*glnB* and *glnK* deletion mutant of Rm1021	Yurgel et al., 2010
Rm1021 *pstC+*	*pstC* repair of Rm1021	This work
Rm1021Δ*glnB*Δ*glnK pstC+*	*glnB* and *glnK* deletion mutant and *pstC* repair of Rm1021	This work
Rm1021 Tn*5*::*phoB*	*phoB* transposon mutant of Rm1021	This work
Rm1021Δ*glnB*Δ*glnK* Tn*5*::*phoB*	*glnB* and *glnK* deletion mutant and *phoB* transposon mutant of Rm1021	This work
*Escherichia coli* S17-1	*pro hsdR recA* RP4-2(Tc::Mu) (Km::Tn*7*)	Simon et al., 1983
Plasmids pK19*mobsacB*	pK19*mob* derivative, *sacB*	Schafer et al., 1994
p2011pstC	pK19*mobsacB* (∼440 bp intergenic region of Rm2011 near SMc02150 and SMc02151)	This work
pJ1700G	pFAJ1700 (XbaI-HindIII *gusA*)	Yurgel et al., 2010
pJ1700G-pGlnII	pJ1700G with 0.5-kb *glnII* promoter	Yurgel et al., 2010
pJ1700G-pGlnA	pJ1700G with 0.5-kb *glnA* promoter	This work
pJ1700G-pPhoX	pJ1700G with 0.5-kb *phoX* promoter	This work

### Genetic Techniques

Standard procedures were used for DNA manipulations (Sambrook, 2001). Tn*5* transposon mutants were generated as described by Yurgel and Kahn (2008) using the non-replicating suicide plasmid pSUP5011 to deliver the transposon to *S. meliloti*. Arbitrary PCR was performed to identify the location of the transposon insertions (Yurgel and Kahn, 2008). Plasmids pJ1700G-pGlnA and pJ1700G-pPhoX were constructed in a manner similar to pJ1700G-pGlnII as described by Yurgel et al. (2010).

Replacement of a wild type allele of *pstC* was carried out through a cloning, conjugation, and transduction method (Labes et al., 1993). In Rm2011, a 438 base pair intergenic segment of DNA (chromosomal position 560651 to 561089) was amplified from an intergenic region upstream of *pstC* (*SMc02145*) between *SMc02151* and *SMc02150*. The amplified DNA was ligated into plasmid pK19*mobsacB* to give p2011*pstC*. Using conjugation, the plasmid was integrated into the Rm2011 chromosome between *SMc02151* and *SMc02150*, marking this region with a neomycin resistance gene contained within the plasmid. Using μ12 bacteriophage-mediated transduction, the integrated plasmid and nearby *pst* operon were delivered from Rm2011 to the Rm1021 strains of interest. Neomycin resistant colonies were selected and streaked for isolation from phage μ12. Visual confirmation of the repair of the *pstC*+ allele was observed on plates containing X-Phos, a substrate for AP that is an indirect method for observing PSR activation that results from the defect in *pstC*. Because of the phosphate transport defect caused by the *pstC* mutation, Rm1021 is normally blue on X-Phos media. White colonies were chosen after transduction. Phage-free colonies were then plated on YMB containing 5% sucrose to select for loss of the p2011*pstC* plasmid by recombination, leaving behind the *pst* operon and surrounding region from Rm2011. The repaired *pstC+* gene was confirmed via DNA sequencing.

### Transduction

Phage-mediated transduction was performed as described by Finan et al. (1984). Phage μ12 was used to deliver DNA into *S. meliloti* strains. Phage lysates were filtered with a 0.2 μ syringe filter to remove bacteria before infecting new cultures. Resulting colonies were selected on the appropriate antibiotic and streaked out until the phage was eliminated (Yurgel et al., 2012).

### Electroporation of *S. meliloti*

Electroporation of *S. meliloti* was based on a protocol by Weigel and Glazebrook (2006) for transformation of *Agrobacterium.* Electrocompetent *S. meliloti* were made by growing 250 mL of cells in LB broth to an A_600_ of ∼0.2. Cells were spun down and washed with cold 10% glycerol four times, each time concentrating the cells in one fifth the volume of glycerol. Cells were resuspended in a final volume of 1 mL and flash frozen with liquid nitrogen before storage at -80°C. 50 μL of electrocompetent *S. meliloti* were incubated for 10 min with ∼2500 ng of plasmid before being electroporated at 2.0 kV in a 2 mm gap length cuvette. Cells were allowed to recover in 1 mL LB broth and grown overnight at 30°C with agitation. The recovered cells were plated on LB media containing the appropriate antibiotic for selection of *S. meliloti* containing the desired plasmid.

### Replication Test

To test growth rate and colony size on Min MOPS media, replication tests were performed as described by Yurgel et al. (2010). Briefly, cells were resuspended to an A_600_ of 0.05. 100 μL of resuspended cells were added to one row of wells of a 96 well plate. Serial 10-fold dilutions were made ending with a 1:10^6^ dilution. A sterile bolt replicator was used to transfer aliquots of each dilution to solid media. Plates were then incubated at 30°C and checked at various times to compare growth between the strains.

### Western Blot

Western blot procedures were carried out according to Yurgel and Kahn (2008) with a few modifications. Cells were grown in high (containing 0.4% NH_4_Cl) or low (containing 0.04% glutamate) nitrogen media for 18 h before being spun down and resuspended in 1 M TE buffer. SDS loading dye was added and proteins were denatured by incubating at 95°C for 20 min. Proteins were separated on a 10% bis-tris acrylamide gel (Invitrogen) then transferred to a nitrocellulose membrane (Whatman) by electrophoresis. GSII was detected using a *S. meliloti* anti-GSII antibody (Shatters et al., 1989). The antibody/protein complex was detected using the Supersignal West Pico Chemiluminescence kit (Thermo Scientific).

### GS Activity

Glutamine synthetase activity was measured by performing the γ-glutamyltransferase assay. Approximate GS activity was quantified based on the formation of γ-glutamylhydroxamate from glutamine and hydroxylamine. Cultures were grown on Minimal MOPS plates (Bardin et al., 1996) with 50 μM KH_2_PO_4_ (low Phosphate) or 2 mM KH_2_PO_4_ (high Phosphate) containing 0.4% NH_4_Cl (nitrogen-sufficient) or 0.04% glutamate (nitrogen-deficient) with succinate as a carbon source for 60 h and then colonies were resuspended in broth of the same composition. GS activity was then performed as described by Somerville and Kahn (1983). Activity units are calculated using A_540_/(mg protein^∗^min).

### β-Glucuronidase Assay

Cultures were grown for 48 h in minimal MOPS media with 50 μM KH_2_PO_4_ (low phosphate) or 2 mM KH_2_PO_4_ (high phosphate) containing 0.4% NH_4_Cl (nitrogen-sufficient) or 0.04% glutamate (nitrogen-deficient). Cultures were diluted 1:40 and grown for 18 h. Expression reactions were run as described by Miller (1972) using 4-nitrophenyl-β-glucuronide (ONPGluc) as the colorimetric substrate. Culture absorbance at 600 nm was measured as well as absorbance at 415 nm, which was due to release of o-nitrophenol. Approximate β-D-glucuronidase activity was calculated using the formula A_415_/(A_600_^∗^min).

### Alkaline Phosphatase Assay

Alkaline phosphatase activity was measured as described by Yurgel et al. (2000). Briefly, cultures were grown on minimal MOPS plates with sufficient or deficient nitrogen and/or phosphate for 72 h at 30°C. Cells were resuspended and washed in 1 M Tris HCl, pH 8 and resuspended again in 1 ml of 1 M Tris HCl. The reaction was started when 0.1 mL of p-nitrophenyl phosphate (pNPP) substrate (Sigma) was added to 0.9 mL of cells and incubated at 30°C for 60 min. Reactions were stopped by addition of 0.1 mL of 1 M KH_2_PO_4_. Absorbance of cell cultures was measured at 600 nm and absorbance of the reaction mix was measured at 420 nm. Activity units are A_420_/(A_600_^∗^min).

## Results

### Alleviation of BK Strain Growth Inhibition by a *phoB*::Tn*5* Transposon Mutation

One of the most striking phenotypes of the BK deletion strain was its very slow growth rate and associated small colony size (Yurgel et al., 2010). *S. meliloti* BK was mutagenized with transposon Tn*5*, allowing the transposon to integrate randomly throughout the genome. Colonies were selected that were larger in size and seven distinct Tn*5* insertion locations were identified in these colonies. Phage-mediated transduction was used to determine if the transposon was associated with faster growth by moving the neomycin resistant transposon into slow-growing BK cells. Of the seven identified locations, only one, containing a Tn*5* insertion in *phoB*, was linked to improved growth of the BK strain, indicating that faster growth by the other six was due to unlinked mutations. The *phoB*::Tn*5* mutation was in the DNA binding/sigma70 interacting C-terminal domain of PhoB, the response regulator in the PSR (**Figure 1C**) (Blanco et al., 2011).

PhoB regulates AP and AP activation is often used as a measure of the PSR and phosphate stress. In order to determine if the phoB mutant was able to activate the PSR, the mutant was placed on LB media containing X-Phos, a substrate for AP that produces a blue pigment when X-Phos is hydrolyzed (Long et al., 1988). Strains containing the *phoB*::Tn*5* mutation produced white colonies on X-Phos media whereas strains with the unrepaired *phoB* were blue. This indicated that the *phoB*::Tn*5* mutant lacked the ability to induce a PSR. The other six faster-growing transposon mutants were blue on X-Phos, suggesting that the spontaneous mutations that resulted in faster growth were not due to mutations inactivating AP or the PSR. This data indicates that mutations in other pathways, not just the PSR, can alter the growth rate of strains deficient in the P_II_ proteins, GlnB and GlnK. Multiple attempts to delete *phoB* were unsuccessful and in the published literature, most *phoB* mutants in *S. meliloti* include a transposon or other mutation at the C-terminal end of the protein similar to the mutant isolated here (Krol and Becker, 2004). While the mutation in *phoB* was initially thought of as a knockout mutation, it may be a protein with altered function instead of a true knockout. The lack of a mutant described in literature as a complete gene deletion or disruption or mutation in the N-terminal region in *S. meliloti* suggests that this protein cannot be knocked out completely and that the truncated version may retain some sort of activity despite its inability to mediate the PSR.

**FIGURE 2:**
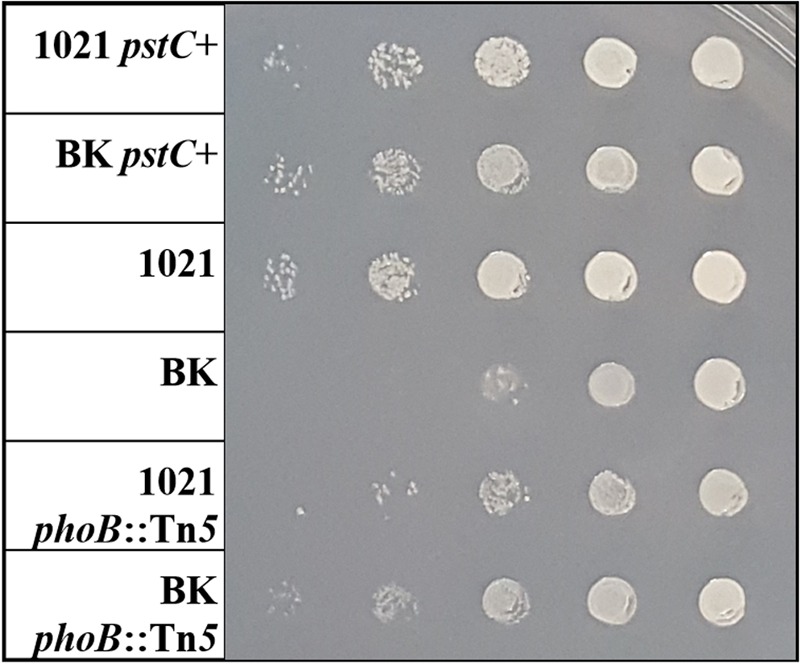
**Replication test of various Rm1021 strains showing growth and colony size at 72 h on Min MOPS Glu with 50 μM KH_2_PO_4_ media.** From left: Rm1021 *pstC+*, BK *pstC+*, Rm1021, BK, Rm1021 *phoB*::Tn*5*, BK *phoB::*Tn*5.* Columns are 1:10 dilutions from top to bottom.

In order to determine the impact of the *phoB*::Tn*5* in a wild type background, the mutation was also transduced into Rm1021. A replication test of the various Rm1021 and BK strains discussed in this paper was performed on Min MOPS media with nitrogen and phosphate sufficient and deficient conditions. **Figure 2** shows a representative plate on nitrogen and phosphate sufficient media after 3 days of growth. All media conditions showed similar growth patterns between strains, but it was most pronounced on the N and P sufficient media. BK grew much more slowly than Rm1021, as described previously (Yurgel et al., 2010). The BK strain containing the *phoB*::Tn*5* mutation grew at a faster rate and produced larger colonies than the BK strain with wild type *phoB*. The BK strains had a smaller colony size than the Rm1021 strains for each pair of mutants (wild type, *pstC*+, *phoB*::Tn*5*), indicating that the absence of the P_II_ genes affected colony size and growth rate in all backgrounds. However, adding either *pstC*+ or *phoB*::Tn*5* to the BK background improved growth, suggesting that activation of the PSR was inhibitory.

### GSII is Downregulated by the *phoB* Mutation

**FIGURE 3:**
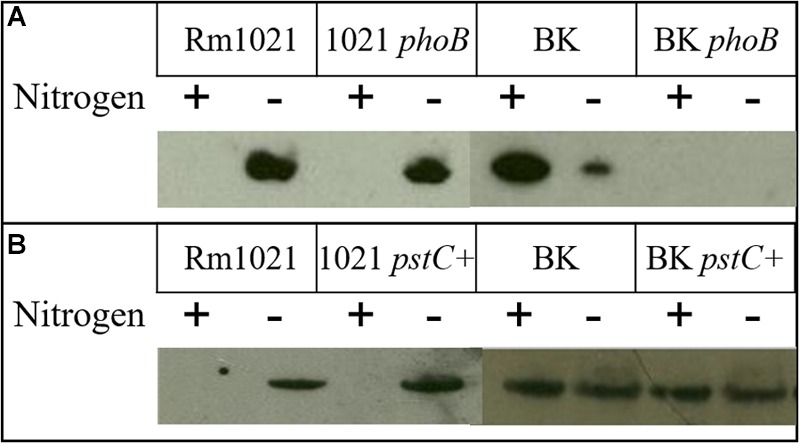
**Western blot of GSII protein abundance in various strains and growth conditions.** Wild type (1021) or BK mutant strains with or without **(A)** the *phoB* transposon mutation or **(B)** a repaired *pstC*+ gene. In the nitrogen row the + or - indicates cultures grown in (+) MMNH_4_+ or (-) MM Glu, respectively.

Another striking phenotype of the BK deletion strain observed previously was the altered abundance of GSII protein during both nitrogen sufficient and deficient conditions (Yurgel et al., 2010). Typically, GSII is only detected during nitrogen stress but its expression in the BK background was very high and actually higher when ammonium was present. To test whether GSII continued to be expressed in the BK strain containing the *phoB*::Tn*5* mutation, the protein was visualized using Western blots (**Figure 3A**; **Supplementary Figure S1**). In Rm1021 *phoB*::Tn*5*, GSII was detected only during nitrogen stress, similar to the pattern of expression of GSII in Rm1021 without the *phoB* mutation. However, the BK *phoB*::Tn*5* mutant did not have any detectable GSII protein when grown in either nitrogen condition, in contrast to what was observed in the BK strain and the wild type. GSII protein levels in *pstC+* strains were similar to the parent strains (**Figure 3B**) in that GSII in Rm1021 and its pstC+ derivative were repressed by ammonium, while GSII was still expressed in BK and its *pstC*+ derivative in the presence of ammonium. These data suggest that GlnB/GlnK and PhoB are both involved in the expression of the GSII protein. Since the original expectation was that the *ΔglnBΔglnK* mutations in BK would block the NSR and expression of GSII, these results are consistent with the idea that expression of GSII in BK is anomalous and that the anomaly depends on PhoB.

The phenotypic differences in the *phoB*::Tn*5* mutant between the Rm1021 and BK backgrounds suggest interactions between the nitrogen and PSRs. This hypothesis also suggests that phosphate availability may affect the NSR and nitrogen may affect the PSR. In addition to exploring the phosphate and nitrogen effects on the NSR and PSR, the *phoB*::Tn*5* mutant strain was characterized further to better understand how GlnB/GlnK and PhoB may be interacting to jointly regulate the two stress responses.

### Phosphate Availability Influences the NSR

To characterize the effects of nitrogen on the PSR, the PSR must be intact so that classical regulation and activation can be observed. Since the *pstC* mutation in Rm1021 partially activated the PSR, the strains were converted to *pstC+* by transduction. Rm1021 without the repaired *pstC* gene was included in some experiments as a control (not shown) to ensure results were consistent with previously published data (Yurgel et al., 2010). The joint effects of nitrogen and phosphate availability on the NSR and PSR were explored in the *pstC+* strains. Additionally, *phoB*::Tn*5* mutant strains were included in some experiments to gather information on how PhoB may be involved in the regulation of the NSR or crosstalk with the P_II_ proteins. The *pstC+* allele also reverses the slow growth characteristic of the BK strain (**Figure 2**), an effect that is consistent with the observed downregulation of the PSR in this strain as the result of improved phosphate uptake. While this change does not disrupt PhoB, it does lead to less PhoB-activated transcription.

Total GS enzymatic activity was used to determine whether the NSR was active during nitrogen limitation in the newly constructed strains and if the level of activity was influenced by phosphate stress. Most rhizobia are unusual because they contain two distinct GS proteins that are regulated by the NSR using circuits that involve the P_II_ proteins. The expression and overall activity of GSI and GSII are upregulated when nitrogen is limited and downregulated when nitrogen is sufficient. It was shown that, compared to wild type Rm1021, total GS activity increased in a strain missing the P_II_ proteins because, while GSII protein was present in Rm1021 only during nitrogen limitation, GSII protein was present in the BK deletion strain at a very high level when ammonium was available and at a lower level when the nitrogen source was glutamate (Yurgel et al., 2012). The observed increase in GSII protein may be related to the observation that, regardless of nitrogen source, all of the detected GSI in the BK strain was present as GSI-AMP, the inactive form of GSI. In Rm1021, GSI, but not GSI-AMP, was detected under nitrogen stressed conditions, reflecting the need for active enzyme (Yurgel et al., 2010). These results are consistent with the need for PII proteins to transduce the signal generated by GlnD that nitrogen is limited.

**FIGURE 4:**
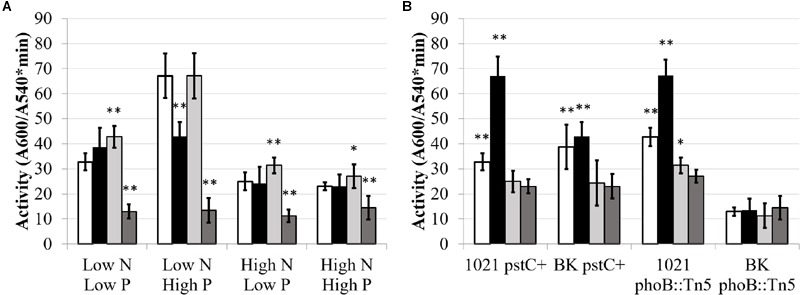
**Total glutamine synthetase activity under different media conditions.** Each bar represents three replicates each from three experiments for a total *n* = 9. Error bars are ±1 SD. Activity units are A_540_/(mg protein^∗^minute). **(A)** Comparison showing differences between strains for each media type on the X-axis. Asterisks are significant differences from Rm1021 *pstC+* for each media condition. ^∗∗^*P* < 0.01; ^∗^*P* < 0.05 as determined by a Student’s *t*-test. White bar is 1021 *pstC+*, black bar is BK *pstC+*, light gray bar is 1021 *phoB*::Tn*5*, dark gray bar is BK *phoB*::Tn*5*. **(B)** Comparison showing differences between media types for each strain on the X-axis. Asterisks are significant differences from High N High P media conditions for each strain. ^∗∗^*P* < 0.01; ^∗^*P* < 0.05 as determined by a Student’s *t*-test. White bar is Low N Low P media, black bar is Low N High P media, light gray bar is High N Low P media, dark gray bar is High N High P media.

**FIGURE 5:**
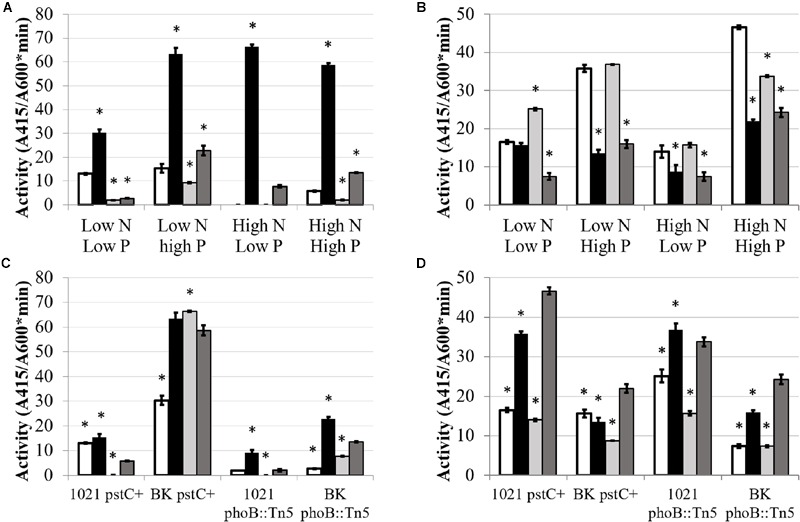
**GusA fusion expression of *glnII* (A,C)** and *glnA* **(B,D)** as a function of media and genetic background. Each bar represents 3 replicates for a total *n* = 3. Error bars are ±1 SD. Activity units are A_415_/(A_600_^∗^minute). **(A,B)** Comparison showing differences between strains for each media type on the X-axis. Asterisks are significant differences from Rm1021 *pstC+* for each media condition as determined by a Student’s *t*-test at *P* < 0.01. White bar is 1021 *pstC+*, black bar is BK *pstC+*, light gray bar is 1021 *phoB*::Tn*5*, dark gray bar is BK *phoB*::Tn*5*. **(C,D)** Comparison showing differences between media type for each strain on the X-axis. Asterisks are significant differences from High N High P for each strain as determined by a Student’s *t*-test at *P* < 0.01. White bar is Low N Low P media, black bar is Low N High P media, light gray bar is High N Low P media, dark gray bar is High N High P media.

Glutamine synthetase activity was affected by phosphate in a wild type background, but not in a P_II_ deletion background. **Figure 4** shows total GS activity in the *pstC+* and *phoB*::Tn*5* strains during high and low nitrogen and phosphate conditions. (The data used to generate **Figures 4** and **5** are included in Supplementary Table S1 in order to permit other comparisons to be made.) When nitrogen was sufficient, phosphate stress did not have a significant effect on total GS activity in any strain. In contrast, when nitrogen was limited, phosphate deficient conditions repressed GS activity by ∼2-fold in Rm1021 *pstC+* and Rm1021 *phoB*::Tn*5* compared to phosphate sufficient conditions. This reduction in GS activity when phosphate availability was limited was not seen in the BK *pstC+* strain. The BK *phoB*::Tn*5* strain had a very low level of total GS activity regardless of the nitrogen or phosphate available, consistent with the idea that both the NSR and PSR were important in enzyme production or activation.

Glutamine synthetase I activity is primarily regulated through adenylylation whereas GSII is primarily regulated through transcription and subsequent translation of the *glnII* gene. GSI and GSII are the protein products of the *glnA* and *glnII* genes. Activity of the *glnA* and *glnII* promoters was examined to see if they responded to phosphate limitation in parallel with GS activity. If phosphate conditions had an effect on the GS activities by influencing transcription, this would be most obvious in the effect on *glnII* expression. Expression of *glnA* and *glnII* were assayed by measuring β-glucuronidase activity generated by the activity of the *glnA* and *glnII* promoters fused to a *gusA* reporter gene in plasmids pJ1700G-pGlnA and pJ1700G-pGlnII.

As expected, *glnII* expression was activated by nitrogen limitation and repressed by ammonium (**Figure 5**). However, the level of this response was regulated by phosphate availability. In Rm1021 *pstC+*, changing phosphate concentration did not lead to a significant change in *glnII* expression when nitrogen was limited, but adding phosphate significantly stimulated *glnII* expression during nitrogen sufficient conditions from an undetectable level to a low but measurable β-glucuronidase activity.

Expression of the *glnII*-*gusA* fusion was strikingly higher in the BK *pstC+* background than in the Rm1021 *pstC+* background, an observation consistent with GSII protein abundance. The level of expression in BK *pstC+* ranged from ∼3-fold higher than in Rm1021 *pstC+* under the “inducing” condition of low N and nearly 100-fold higher in high N where *glnII* expression in Rm1021 *pstC+* was almost completely repressed. The BK *pstC+* strain had reduced activity during phosphate limitation compared to sufficiency when nitrogen was limited, demonstrating a response to phosphate levels under conditions when *glnII* expression is typically induced. Introducing the *phoB*::Tn5 mutation substantially lowered *glnII-gusA* expression in both the wild type and BK backgrounds under each of the media conditions tested. The addition of phosphate raised the level of expression of *glnII-gusA* in both Rm1021 and BK with the *phoB*::Tn*5* mutation indicating that GSII expression was induced by phosphate in the absence of a proper PhoB regulatory protein.

Unlike GSII, GSI is regulated post-translationally by adenylylation. In rhizobia, *glnA* transcription is not as significant in controlling the amount of GSI activity present and the effect of nitrogen concentration on *glnA*-*gusA* expression observed here was not as dramatic as the effect on *glnII-gusA* (**Figure 5**). Raising phosphate increased the expression of *glnA-gusA* for both Rm1021 *pstC+* and BK *pstC+* when nitrogen was available but only Rm1021 *pstC+* had significantly higher expression when nitrogen was limiting. The presence or absence of the *phoB*::Tn*5* mutation did not have a strong effect on the expression of *glnA-gusA* under most conditions but, for both 1021 *phoB*::Tn*5* and BK *phoB*::Tn*5*, phosphate increased *glnA-gusA* expression by about twofold when phosphate was higher under similar nitrogen conditions.

### Effects of Nitrogen on PSR

**FIGURE 6:**
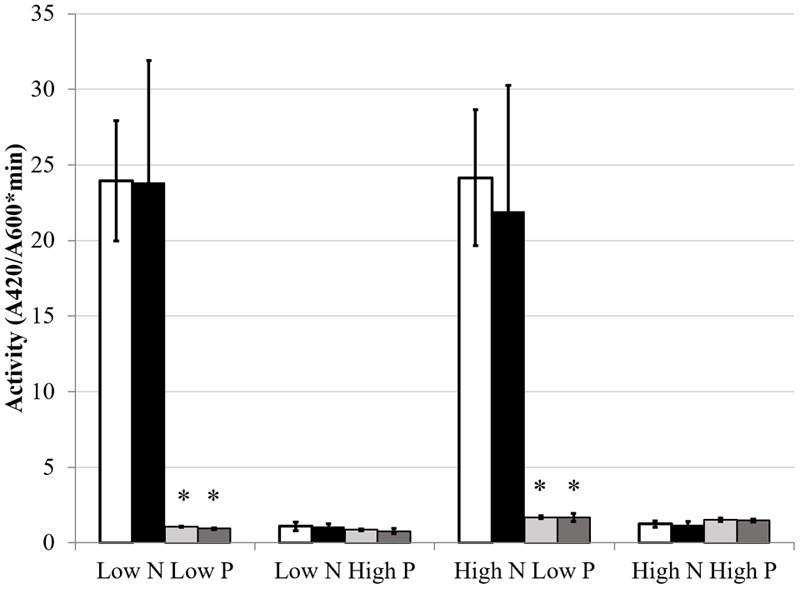
**Alkaline phosphatase activity under different media conditions.** Each bar represents three replicates each from three independent experiments for a total *n* = 9. Error bars are ±1 SD. Activity units are A_420_/(A_600_^∗^minute). In all cases, asterisks are significant differences from Rm1021 *pstC+* for that media condition as determined by a Student’s *t*-test at *P* < 0.01. White bar is 1021 *pstC+*, black bar is BK *pstC+*, light gray bar is 1021 *phoB*::Tn*5*, dark gray bar is BK *phoB*::Tn*5*.

**FIGURE 7:**
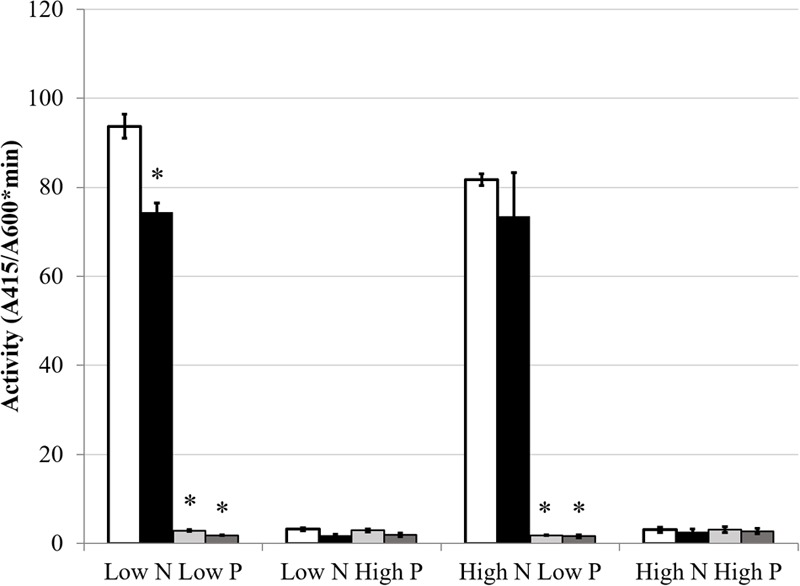
**GusA fusion expression of *phoX*.** Each bar represents three replicates each from two independent experiments for a total *n* = 6. Error bars are ±1 SD. Activity units are A_415_/(A_600_^∗^minute). In all cases, asterisks are significant differences from Rm1021 *pstC+* for that media condition as determined by a Student’s *t*-test at *P* < 0.01. White bar is 1021 *pstC+*, black bar is BK *pstC+*, light gray bar is 1021 *phoB*::Tn*5*, dark gray bar is BK *phoB*::Tn*5*.

The GS activity and expression results indicated that phosphate status affected aspects of the NSR. The hypothesis that nitrogen conditions would affect the PSR was also explored. As discussed previously, AP activity is an index of the PSR that is typically induced by low phosphate conditions. AP expression and activity were measured to determine if nitrogen limitation would influence the production of AP. The classical activation pattern was observed and displayed a very definite on/off activity pattern in the Rm1021 *pstC+* strain when grown in low or high phosphate, respectively. When the cells were *pstC+* in either wild type Rm1021 or the BK deletion strain, nitrogen limitation had no significant effect on the activity of AP (**Figure 6**). *phoX*, which codes for the major AP in Rm1021, is upregulated during phosphate limitation in a PhoB-dependent manner (Zaheer et al., 2009). Since phosphatases are typically inhibited by phosphate (Fernley and Walker, 1967), a *gusA* reporter fusion to *phoX* was constructed and introduced into the mutant strains to measure a reporter activity based on expression of the *phoX* promoter. The activity of β-glucuronidase from the *phoX-gusA* fusion mimicked the activity levels of AP for each strain and condition (**Figure 7**). The *pstC+* strains showed increased expression during phosphate limitation and very minimal expression when the phosphate level was sufficient, but changing the nitrogen availability had no effect. The *phoB*::Tn*5* mutant strains had minimal expression of the *phoX*-*gusA*, regardless of phosphate or nitrogen levels. These data show nitrogen status was not having an effect on AP expression or activity in addition to the known regulation by phosphate availability. When any of these strains carried the *phoB*::Tn*5* transposon insertion, they had very low AP or *phoX-gusA* derived β-glucuronidase activity, regardless of nitrogen or phosphate conditions. This pattern of expression would be expected if the *phoB* gene was mutated, *phoX* was regulated by PhoB and the *phoB*::Tn*5* mutation was not leaky in its control of the PSR. This implies that the phosphate responsiveness of both *glnA* and *glnII* expression was not due to some residual PhoB activity and that another level of phosphate regulation in *S. meliloti* appeared to be involved.

## Discussion

The persistence of rhizobia in various soil types is crucial for BNF to occur during symbiosis with plants. Responding to various stresses and nutrient limitations in soil has led to the development of stress response regulatory pathways by bacteria. The nitrogen and PSRs of *S. meliloti* have been characterized independently, and recent studies have indicated a coordination of the stress responses. It had been shown that a mutant lacking the GlnB and GlnK P_II_ proteins, which mediate the NSR, has a striking slow growth phenotype. Faster growing pseudorevertants occur at a significant rate in the BK strain and in order to understand which genes and proteins are involved in the pseudoreversion, transposon mutagenesis was performed. The mutagenesis screening led to the identification of a *phoB*::Tn*5* mutation that could generate faster growing colonies.

In *R. rubrum*, the slow growth phenotype of a P_II_ deletion strain was alleviated by additional mutations that each led to a decrease in overall GS activity (Zhang et al., 2006). Consistent with this, GSII protein was no longer detected in either nitrogen condition in the BK *phoB*::Tn*5* mutant strain, in contrast to the elevated level of GSII found in the BK mutant. This aspect of the *phoB* phenotype was specific to the mutant that lacked P_II_ proteins—the *phoB*::Tn*5* mutation did not affect the pattern of GSII protein expression in Rm1021. It was previously shown that a group of PSR-regulated genes had altered expression in the absence of the P_II_ proteins (Yurgel et al., 2013). The finding that a *phoB* mutation alleviated the growth inhibition in the BK strains led us to explore the hypothesis that the NSR may be affected by phosphate as well as nitrogen, and that nitrogen may be affecting the PSR in addition to phosphate. The effect of phosphate on the NSR was investigated by evaluation of GS genes expression and enzymatic activity and the effect of nitrogen on the PSR was investigated through the AP gene and protein activity.

In addition to the well-characterized nitrogen regulation of the GS enzymes, phosphate availability also affected *S. meliloti* GS expression and activity. The importance of the P_II_ proteins in proper regulation of total GS activity was reinforced as well. In the wild type Rm1021 background, phosphate limitation repressed GS activity in both the *pstC+* and *phoB*::Tn*5* strains when nitrogen was limited. This indicated that, while GS activity depended on nitrogen conditions and regulation involving the P_II_ proteins, phosphate stress further regulated GS activity and suggested the involvement of the P_II_ proteins with the PSR, perhaps through a phosphate-responsive transcription factor. Since Rm1021 *pstC+* and Rm1021 *phoB*::Tn*5* show similar repression patterns of total GS activity, PhoB is unlikely to be involved in the low phosphate repression of GS activity—if it were, this repression should be abolished in the *phoB*::Tn*5* strain. The BK *pstC+* strain displayed the unusual increased GS activity during limited nitrogen, but not during nitrogen sufficient conditions. However, it was not responsive to changing phosphate levels. The consistent low GS activity in the BK *phoB*::Tn*5* strain suggested that both BK and PhoB were required for the proper activation of GS. This phenotype coincided with the changes observed in GSII protein as detected via Western blot indicating some sort of interaction or coordinated regulation between GlnB/GlnK and PhoB.

PhoB is the response regulator of the two component PhoR/PhoB system (**Figure 1**). PhoR phosphorylates PhoB in response to low phosphate conditions, converting PhoB into the more transcriptionally active PhoB-P state. A mutation in PhoR would decrease PhoB-P. A small amount of PhoB could potentially be phosphorylated by small molecules (Wanner and Wilmes-Riesenberg, 1992), but the low level of phosphorylation would likely not be sufficient to induce the necessary response to phosphate stress. We have not investigated whether the lack of PhoB activation due to a PhoR knockout would produce similar phenotypes as the *phoB*::Tn*5* mutant in regard to its regulation of the PSR. However, it is possible that regulatory crosstalk between two component systems is occurring, where PhoR-P is activating NtrC in the absence of PhoB, its cognate response regulator (Verhamme et al., 2002).

In wild type Rm1021, phosphate concentration affected *glnII* expression when nitrogen was available—low phosphate nearly abolished *glnII* expression when nitrogen was sufficient. One potential rationale is that the cell was allocating resources to the PSR when nitrogen was present and the cell did not need to increase the level of GS activity to assimilate sufficient nitrogen. When nitrogen and phosphate are both limited, cells must cope with these stresses simultaneously and may prioritize its response. It is likely the cell continues to commit resources to both stress responses instead of allowing one to function at a high level while the other is severely inhibited by a lack of resources. Another potential explanation for the dual regulation of GS by both nitrogen and phosphate could be that the cell was trying to balance the nitrogen and phosphate pools within the cell. Nucleic acid synthesis requires inputs of both nitrogen and phosphate and an imbalance in the resource pools could be inhibitory. Protein synthesis is less affected by phosphate availability since, while ATP and phosphate are needed at several steps in the process, phosphate is not directed into a sink by the process. The lack of a recognizable Pho box (Yuan et al., 2006b) upstream of *glnII* suggests that PhoB is not directly binding to the *glnII* promoter region. This idea is supported by the increased expression in response to additional phosphate that occurs even when PhoB is absent. The *ntrB/ntrC* promoter also lacks a recognizable Pho box, suggesting that PhoB is not binding in this region and affecting *glnII* expression by directly regulating *ntrB/ntrC* expression. In *Streptomyces coelicolor*, there is direct interaction between PhoP, the response regulator in a two component PSR system, and some promoters involved in its NSR through binding of PhoP at partial Pho box sequences (Rodríguez-García et al., 2009).

Previous studies demonstrated that *glnII* expression was increased in BK deletion strains. This result was explained by the need for a P_II_ protein to dephosphorylate NtrC-P (Yurgel et al., 2010) so that NtrC remains phosphorylated and induces *glnII* expression. We found that *glnII* expression is elevated in the BK *pstC+* strain in all media conditions, including in the presence of ammonium. One explanation for increased GSII in BK during nitrogen limitation is that the increased GSII compensates for the adenylylation and inactivation of GSI (Yurgel et al., 2010). Another potential explanation is that GSI is always modified in the BK deletion as a result of the very high levels of GSII protein. The unexpected connection reported here is that when PhoB is mutated in the BK strains, GSII expression and protein levels are drastically reduced. This observation supports the idea that GlnB/GlnK and PhoB mediate a coordinated response to some nutrient conditions.

The data show that the PSR, as measured by AP induction or activity, was not affected by nitrogen availability. This suggests that the cell prioritizes phosphate acquisition during phosphate limitation and concurrent nitrogen deficiency was not affecting activation of the PSR. Due to the low level of AP activity and expression when phosphate was available, there was essentially no opportunity to observe an impact of nitrogen deficiency on expression or activity of AP as was observed when *glnII* expression was altered by phosphate limitation. Yurgel et al. (2013) found that *phoX* was induced in the BK deletion strain and by nitrogen limitation in high phosphate media. In the *phoX-gusA* reporter fusion experiments described above, expression levels were low in high phosphate medium. The sensitivity of the Yurgel et al. (2013) transcriptome study may have allowed the detection of a threefold change in mRNA that was not observable as a comparable change in the level of the PhoX-GusA fusion protein. The lack of activity and expression of AP in the *phoB*::Tn*5* strains demonstrated that the mutation was eliminating the ability of PhoB to induce PSR.

This coordinated response of GS to both nitrogen and phosphate is leading to the exploration of the interaction and dual regulation of the NSR by both nitrogen and phosphate at a deeper level. Global transcription changes in response to nitrogen and phosphate nutrient deficiencies are being characterized to determine how *S. meliloti* gene expression is regulated by multiple stresses and which cellular processes are impacted the most. A closer examination of PSR and NSR transcript changes is being conducted to determine if there are genes that are regulated by nitrogen or phosphate, respectively. Additionally, transcript changes in the BK background are being examined during one or both nutrient stresses to help understand the interaction between the P_II_ genes and the PSR. Further research might include examining how post-translationally modified protein abundance may be altered by the various media conditions since signal transduction and enzyme activity are known to be important in the NSR. While the data presented here focus on the NSR and PSR, an important question highlighted by this research is whether there are interactions between other nutritional stress responses and, if so, whether these might be mediated by a well-defined integrative mechanism. Understanding how multiple stresses affect each pathway and ultimately gaining insight into how stress responses are coordinately regulated in bacteria may be crucial to understanding which strains are best suited to various soil conditions. The limitation of nitrogen, phosphate, or both in the soil influences the microbial community and can have significant effects on relative levels of bacteria and fungi present (Vitousek et al., 2010; Leff et al., 2015; Fanin et al., 2016). As nutrient levels change and may shift from one limitation to co-limitation, the microbial populations shift as well. A better understanding of how nitrogen and phosphate availability affects both the NSR and PSR can serve to facilitate the selection of strains that may be best suited for certain soil types and the change in nutrients across seasons. In the context of symbiotic nitrogen fixation, response to limiting factors in the soil may determine how rapidly a strain can respond to the opportunity presented by a legume root and be important in competitiveness and an increased crop yield.

## Author Contributions

Study conception and design: KH, MK, SY; acquisition of data: KH, MM; analysis and interpretation of data: KH, MK, SY; drafting of manuscript: KH, MK; critical revision: KH, MK, SY.

## Conflict of Interest Statement

The authors declare that the research was conducted in the absence of any commercial or financial relationships that could be construed as a potential conflict of interest.

## Supplementary Material

FIGURE S1 **(A)** GSII Western blots from **Figure 3** with Coomassie stained protein gel. Ladder on far right of stained gel, followed by lanes corresponding to the GSII Western conditions and strains as indicated. **(B)** GSII Western blots from **Figure 3** with Coomassie stained membranes to show loaded protein.
