# Supplementary figures and images for: Interaction between Nitrogen and Phosphate Stress Responses in Sinorhizobium meliloti
Source: Front Microbiol. 2016 Nov 30;7:1928. doi: 10.3389/fmicb.2016.01928 (PMC5127829; doi:10.3389/fmicb.2016.01928)

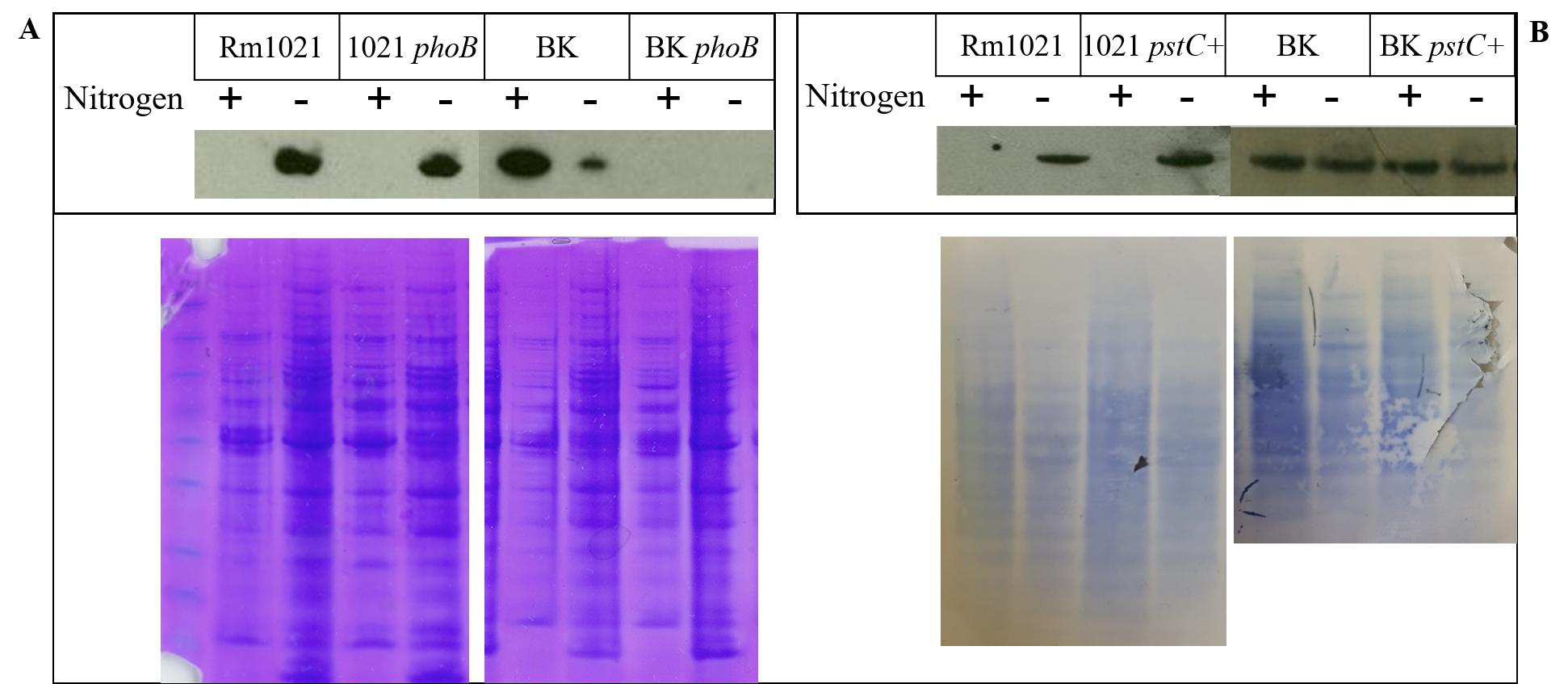

Supplement: FIGURE S1 — (A) GSII Western blots from Figure 3 with Coomassie stained protein gel. Ladder on far right of stained gel, followed by lanes corresponding to the GSII Western conditions and strains as indicated. (B) GSII Western blots from Figure 3 with Coomassie stained membranes to show loaded protein. [file Image_1.TIF]
