# Supplementary material for: Interaction between Nitrogen and Phosphate Stress Responses in Sinorhizobium meliloti
Source: Front Microbiol. 2016 Nov 30;7:1928. doi: 10.3389/fmicb.2016.01928 (PMC5127829; doi:10.3389/fmicb.2016.01928)
Supplement: Supplementary file 2 [file Table_1.DOCX]

Supplemental Table 1: Raw values with significance for GS activity and *glnA::gusA* and *glnII::gusA* fusion expression. Values are ± one standard deviation. Asterisks indicates values for each assay that are significantly different than 1021 for the specified media condition as determined by a Student’s t-test where **=P<0.01 and *=P<0.05.

|  | Media type | 1021 pstC+ | BK pstC+ | 1021 phoB::Tn5 | BK phoB::Tn5 |
| --- | --- | --- | --- | --- | --- |
| GS Activity | Low N Low P | 32.8 ± 3.5 | 38.8 ± 7.7 | 42.8 ± 4.3** | 13.0 ± 2.8** |
|  | Low N High P | 67.2 ± 8.9 | 43.0 ± 5.7** | 67.2 ± 9.0 | 13.5 ± 4.9** |
|  | High N Low P | 25.0 ± 3.6 | 24.4 ± 6.5 | 31.5 ± 3.1** | 11.3 ± 2.5** |
|  | High N High P | 23.1 ± 1.7 | 23.0 ± 4.7 | 27.1 ± 4.8* | 14.5 ± 4.7** |
| *glnII::gusA* expression | Low N Low P | 13.0 ± 0.3 | 30.3 ± 1.2** | 2.0 ± 0.1** | 2.7 ± 0.1** |
|  | Low N High P | 15.4 ± 1.8 | 63.3 ± 2.5** | 9.3 ± 0.2** | 22.7 ± 2.1** |
|  | High N Low P | 0 | 66.3 ± 0.9** | 0 | 7.7 ± 0.5** |
|  | High N High P | 5.8 ± 0.3 | 58.6 ± 0.9** | 2.0 ± 0.2** | 13.6 ± 0.2** |
| *glnA::gusA* expression | Low N Low P | 16.5 ± 0.5 | 15.7 ± 1.0 | 25.1 ± 1.7** | 7.4 ± 0.5** |
|  | Low N High P | 35.8 ± 0.4 | 13.6 ± 0.4** | 36.9 ± 0.7 | 15.9 ± 0.4** |
|  | High N Low P | 14.0 ± 0.3 | 8.8 ± 0.1** | 15.7 ± 0.5 | 7.4 ± 0.3** |
|  | High N High P | 46.6 ± 0.9 | 22.0 ± 1.1** | 33.8 ± 1.1** | 24.3 ± 1.2** |
